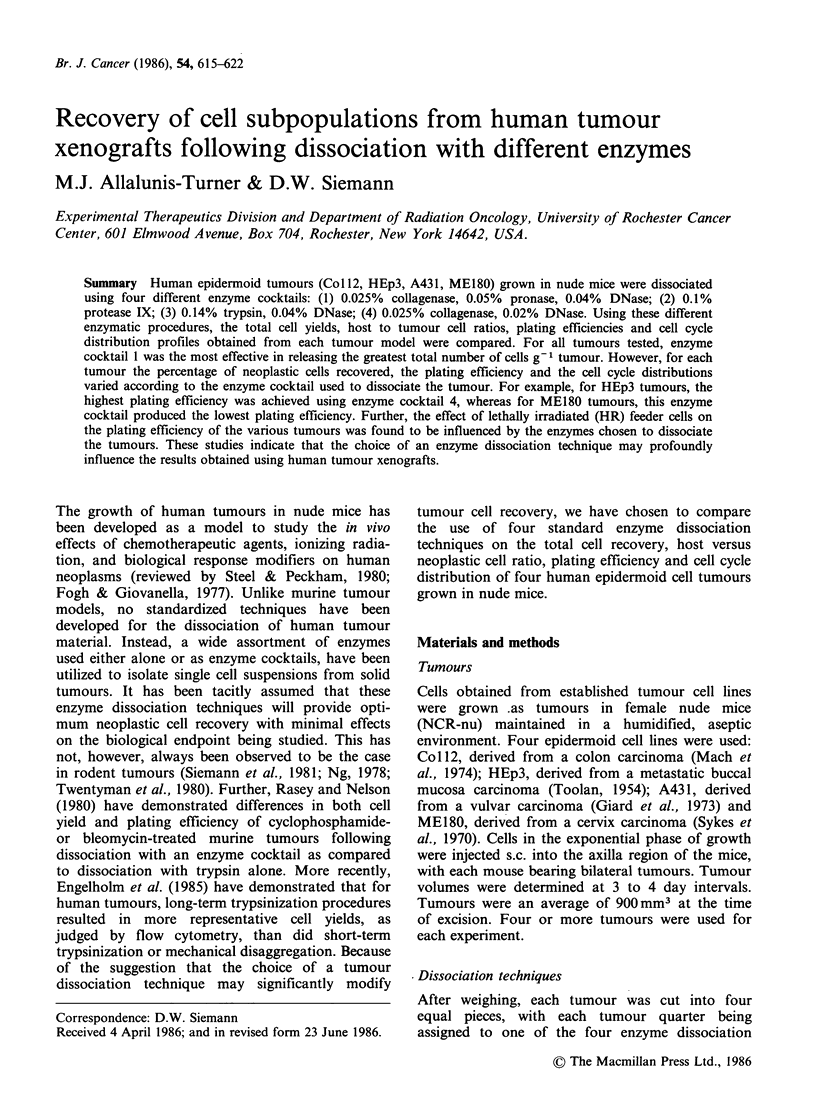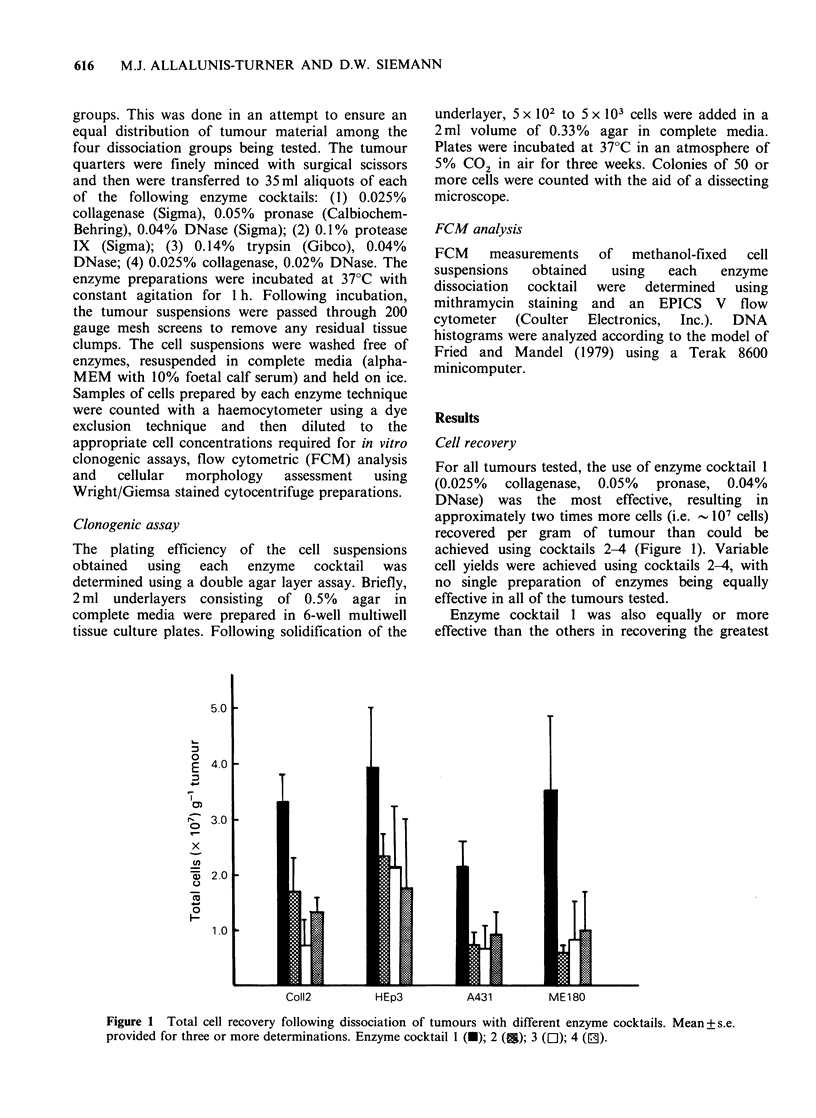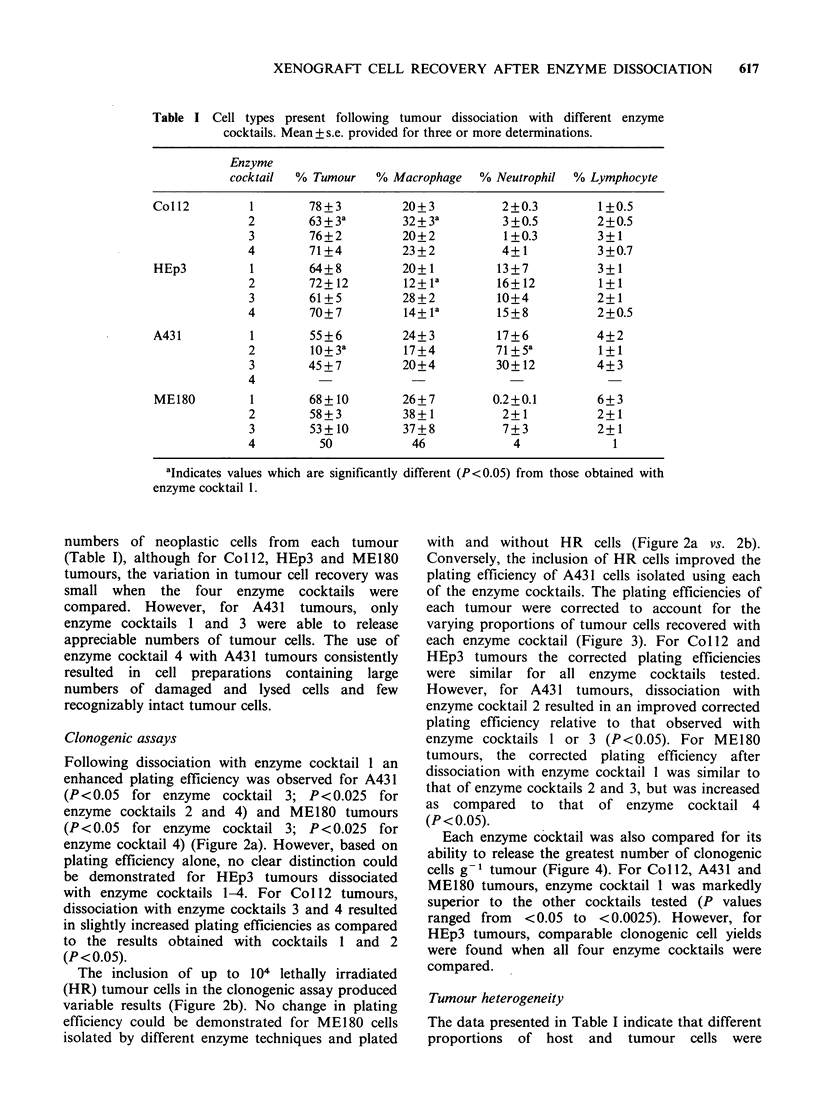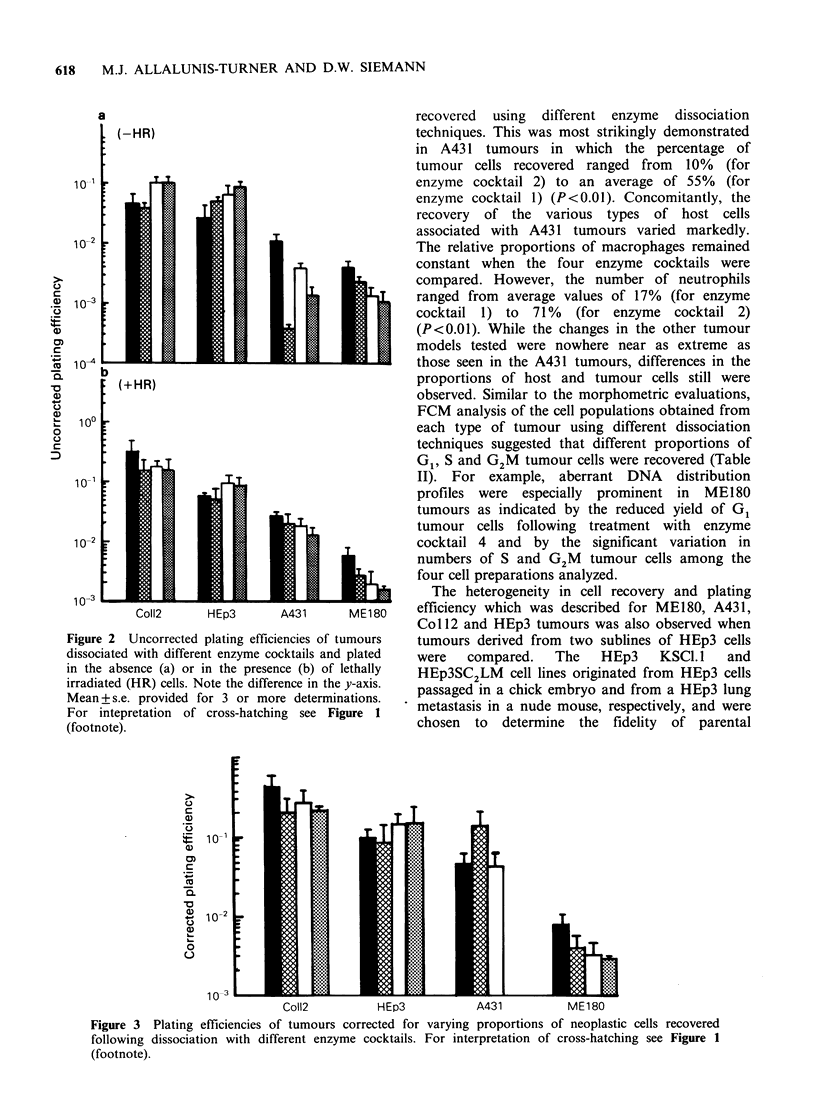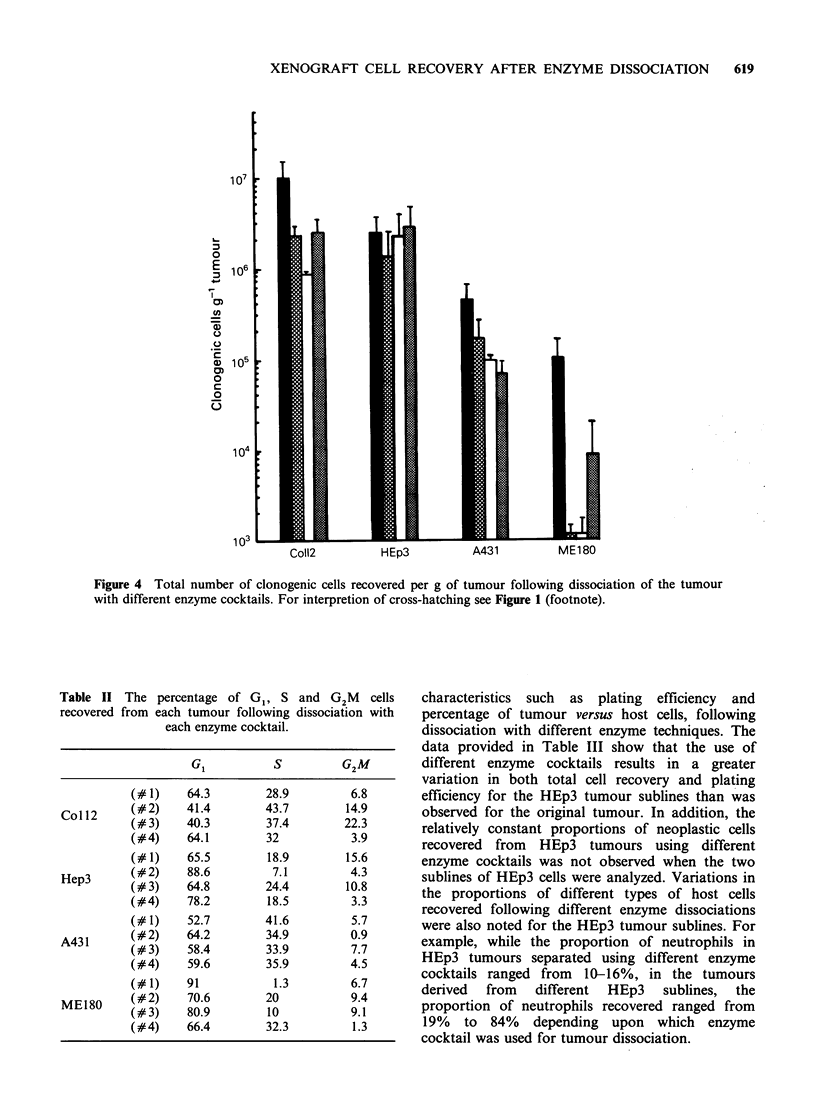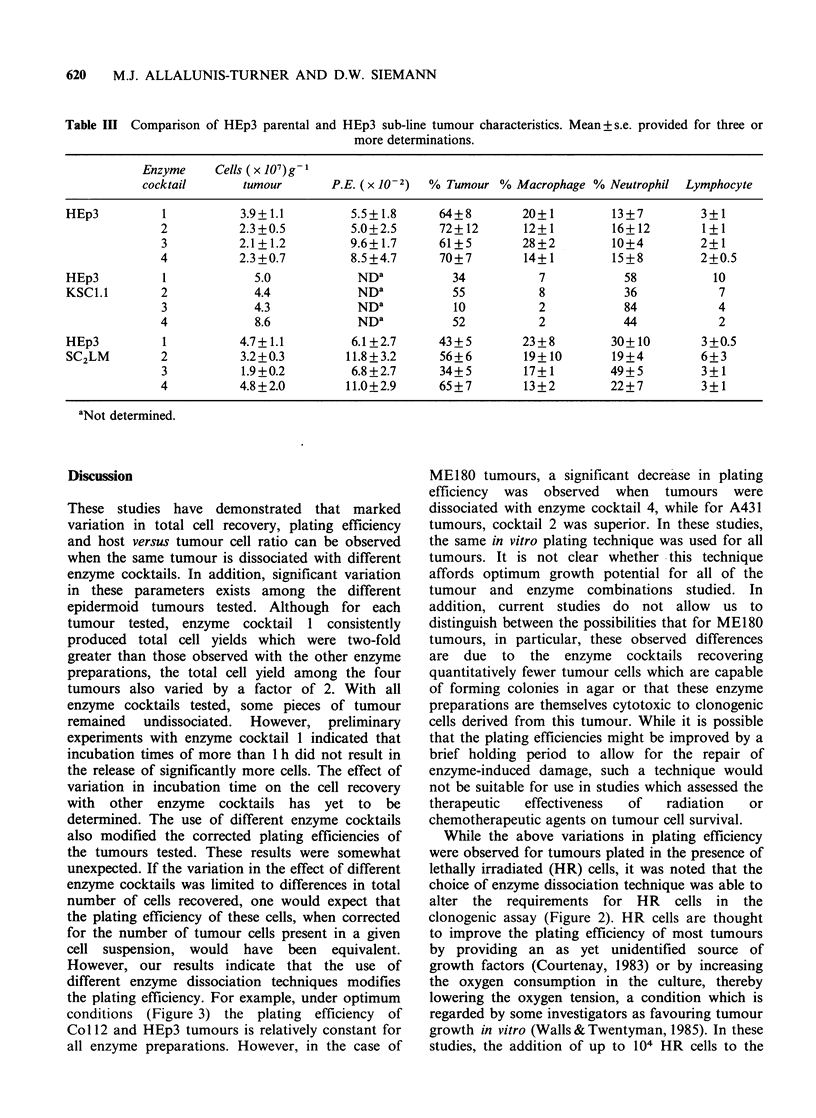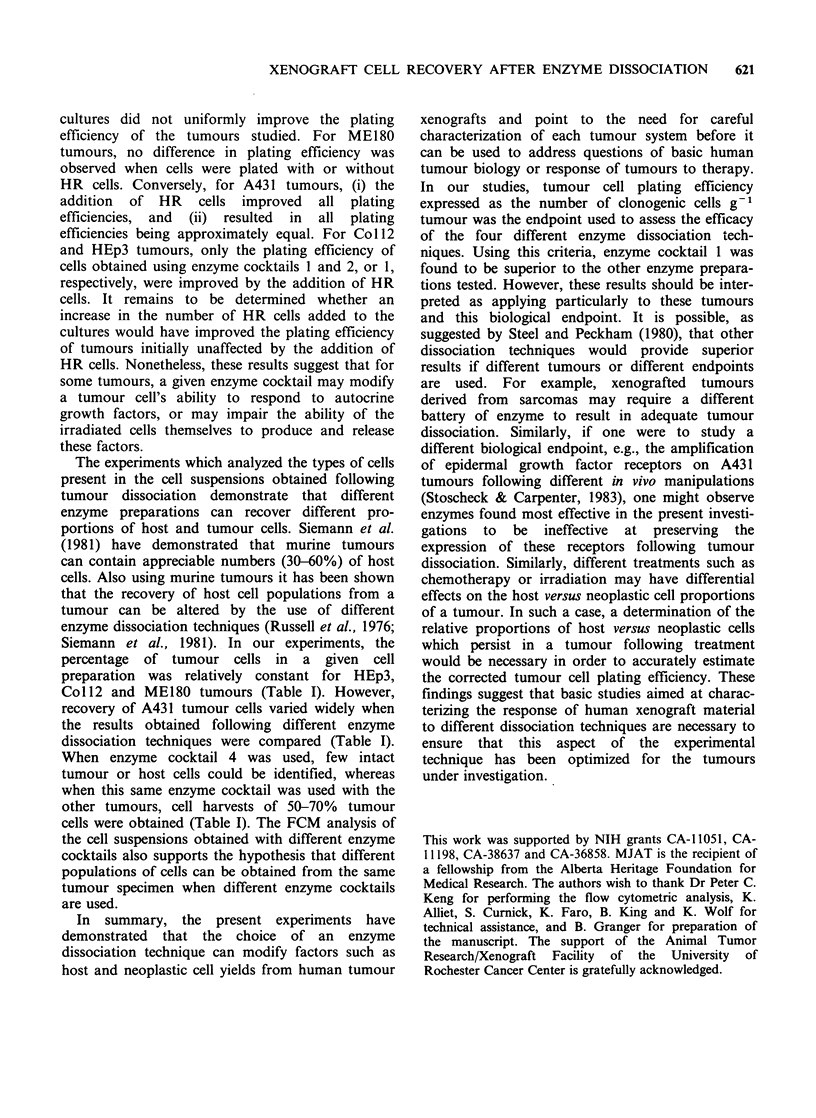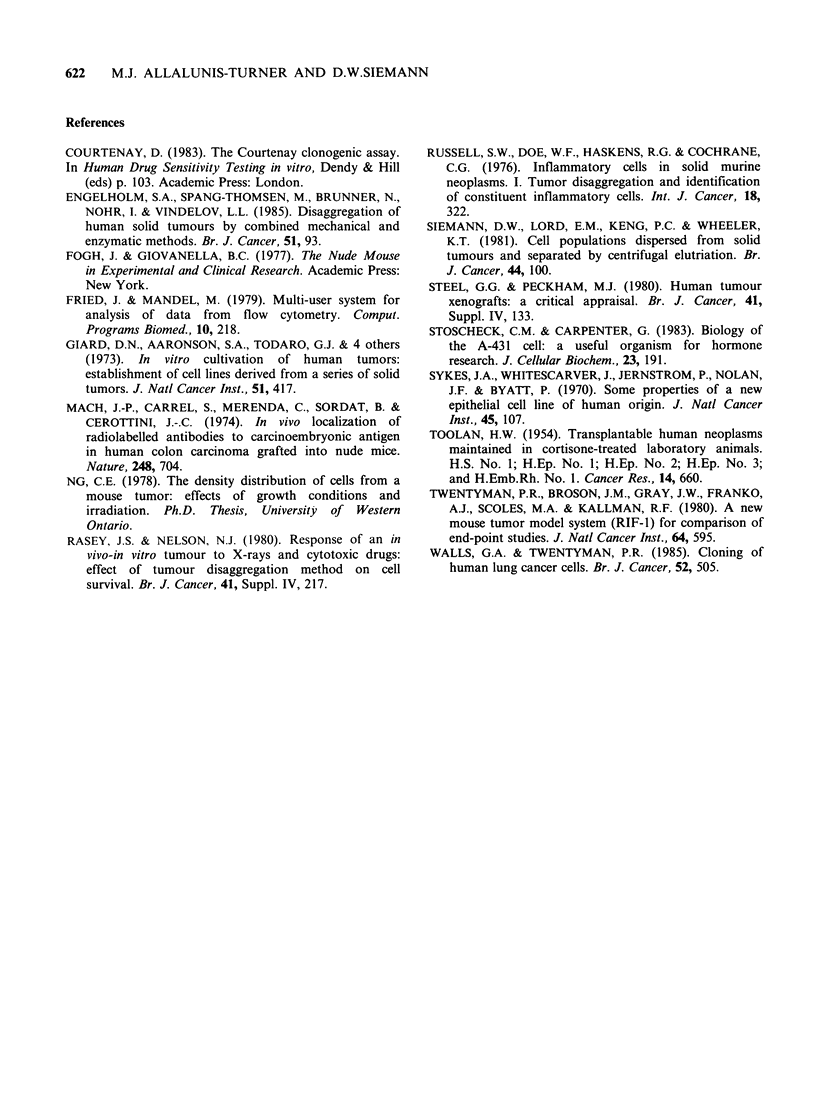# Recovery of cell subpopulations from human tumour xenografts following dissociation with different enzymes.

**DOI:** 10.1038/bjc.1986.217

**Published:** 1986-10

**Authors:** M. J. Allalunis-Turner, D. W. Siemann

## Abstract

Human epidermoid tumours (Co112, HEp3, A431, ME180) grown in nude mice were dissociated using four different enzyme cocktails: 0.025% collagenase, 0.05% pronase, 0.04% DNase; 0.1% protease IX; 0.14% trypsin, 0.04% DNase; 0.025% collagenase, 0.02% DNase. Using these different enzymatic procedures, the total cell yields, host to tumour cell ratios, plating efficiencies and cell cycle distribution profiles obtained from each tumour model were compared. For all tumours tested, enzyme cocktail 1 was the most effective in releasing the greatest total number of cells g-1 tumour. However, for each tumour the percentage of neoplastic cells recovered, the plating efficiency and the cell cycle distributions varied according to the enzyme cocktail used to dissociate the tumour. For example, for HEp3 tumours, the highest plating efficiency was achieved using enzyme cocktail 4, whereas for ME180 tumours, this enzyme cocktail produced the lowest plating efficiency. Further, the effect of lethally irradiated (HR) feeder cells on the plating efficiency of the various tumours was found to be influenced by the enzymes chosen to dissociate the tumours. These studies indicate that the choice of an enzyme dissociation technique may profoundly influence the results obtained using human tumour xenografts.


*Br. J. Cancer* (1986), **54**, 615–622

### Recovery of cell subpopulations from human tumour xenografts following dissociation with different enzymes

M.J. Allalunis-Turner & D.W. Siemann

*Experimental Therapeutics Division and Department of Radiation Oncology, University of Rochester Cancer Center, 601 Elmwood Avenue, Box 704, Rochester, New York 14642, USA.*

**Summary** Human epidermoid tumours (Co112, HEp3, A431, ME180) grown in nude mice were dissociated using four different enzyme cocktails: (1) 0.025% collagenase, 0.05% pronase, 0.04% DNase; (2) 0.1% protease IX; (3) 0.14% trypsin, 0.04% DNase; (4) 0.025% collagenase, 0.02% DNase. Using these different enzymatic procedures, the total cell yields, host to tumour cell ratios, plating efficiencies and cell cycle distribution profiles obtained from each tumour model were compared. For all tumours tested, enzyme cocktail 1 was the most effective in releasing the greatest total number of cells $g^{-1}$ tumour. However, for each tumour the percentage of neoplastic cells recovered, the plating efficiency and the cell cycle distributions varied according to the enzyme cocktail used to dissociate the tumour. For example, for HEp3 tumours, the highest plating efficiency was achieved using enzyme cocktail 4, whereas for ME180 tumours, this enzyme cocktail produced the lowest plating efficiency. Further, the effect of lethally irradiated (HR) feeder cells on the plating efficiency of the various tumours was found to be influenced by the enzymes chosen to dissociate the tumours. These studies indicate that the choice of an enzyme dissociation technique may profoundly influence the results obtained using human tumour xenografts.

The growth of human tumours in nude mice has been developed as a model to study the *in vivo* effects of chemotherapeutic agents, ionizing radiation, and biological response modifiers on human neoplasms (reviewed by Steel & Peckham, 1980; Fogh & Giovanella, 1977). Unlike murine tumour models, no standardized techniques have been developed for the dissociation of human tumour material. Instead, a wide assortment of enzymes used either alone or as enzyme cocktails, have been utilized to isolate single cell suspensions from solid tumours. It has been tacitly assumed that these enzyme dissociation techniques will provide optimum neoplastic cell recovery with minimal effects on the biological endpoint being studied. This has not, however, always been observed to be the case in rodent tumours (Siemann *et al.*, 1981; Ng, 1978; Twentyman *et al.*, 1980). Further, Rasey and Nelson (1980) have demonstrated differences in both cell yield and plating efficiency of cyclophosphamide- or bleomycin-treated murine tumours following dissociation with an enzyme cocktail as compared to dissociation with trypsin alone. More recently, Engelholm *et al.* (1985) have demonstrated that for human tumours, long-term trypsinization procedures resulted in more representative cell yields, as judged by flow cytometry, than did short-term trypsinization or mechanical disaggregation. Because of the suggestion that the choice of a tumour dissociation technique may significantly modify tumour cell recovery, we have chosen to compare the use of four standard enzyme dissociation techniques on the total cell recovery, host versus neoplastic cell ratio, plating efficiency and cell cycle distribution of four human epidermoid cell tumours grown in nude mice.

Correspondence: D.W. Siemann
Received 4 April 1986; and in revised form 23 June 1986.

### Materials and methods

### *Tumours*

Cells obtained from established tumour cell lines were grown as tumours in female nude mice (NCR-nu) maintained in a humidified, aseptic environment. Four epidermoid cell lines were used: Co112, derived from a colon carcinoma (Mach *et al.*, 1974); HEp3, derived from a metastatic buccal mucosa carcinoma (Toolan, 1954); A431, derived from a vulvar carcinoma (Giard *et al.*, 1973) and ME180, derived from a cervix carcinoma (Sykes *et al.*, 1970). Cells in the exponential phase of growth were injected s.c. into the axilla region of the mice, with each mouse bearing bilateral tumours. Tumour volumes were determined at 3 to 4 day intervals. Tumours were an average of 900 $mm^3$ at the time of excision. Four or more tumours were used for each experiment.

### *Dissociation techniques*

After weighing, each tumour was cut into four equal pieces, with each tumour quarter being assigned to one of the four enzyme dissociation

© The Macmillan Press Ltd., 1986

groups. This was done in an attempt to ensure an equal distribution of tumour material among the four dissociation groups being tested. The tumour quarters were finely minced with surgical scissors and then were transferred to 35 ml aliquots of each of the following enzyme cocktails: (1) 0.025% collagenase (Sigma), 0.05% pronase (Calbiochem-Behring), 0.04% DNase (Sigma); (2) 0.1% protease IX (Sigma); (3) 0.14% trypsin (Gibco), 0.04% DNase; (4) 0.025% collagenase, 0.02% DNase. The enzyme preparations were incubated at 37°C with constant agitation for 1 h. Following incubation, the tumour suspensions were passed through 200 gauge mesh screens to remove any residual tissue clumps. The cell suspensions were washed free of enzymes, resuspended in complete media (alpha-MEM with 10% foetal calf serum) and held on ice. Samples of cells prepared by each enzyme technique were counted with a haemocytometer using a dye exclusion technique and then diluted to the appropriate cell concentrations required for *in vitro* clonogenic assays, flow cytometric (FCM) analysis and cellular morphology assessment using Wright/Giemsa stained cytocentrifuge preparations.

*Clonogenic assay*

The plating efficiency of the cell suspensions obtained using each enzyme cocktail was determined using a double agar layer assay. Briefly, 2 ml underlayers consisting of 0.5% agar in complete media were prepared in 6-well multiwell tissue culture plates. Following solidification of the underlayer, $5 \times 10^2$ to $5 \times 10^3$ cells were added in a 2 ml volume of 0.33% agar in complete media. Plates were incubated at 37°C in an atmosphere of 5% $CO_2$ in air for three weeks. Colonies of 50 or more cells were counted with the aid of a dissecting microscope.

*FCM analysis*

FCM measurements of methanol-fixed cell suspensions obtained using each enzyme dissociation cocktail were determined using mithramycin staining and an EPICS V flow cytometer (Coulter Electronics, Inc.). DNA histograms were analyzed according to the model of Fried and Mandel (1979) using a Terak 8600 minicomputer.

### Results

*Cell recovery*

For all tumours tested, the use of enzyme cocktail 1 (0.025% collagenase, 0.05% pronase, 0.04% DNase) was the most effective, resulting in approximately two times more cells (i.e. $\sim 10^7$ cells) recovered per gram of tumour than could be achieved using cocktails 2–4 (Figure 1). Variable cell yields were achieved using cocktails 2–4, with no single preparation of enzymes being equally effective in all of the tumours tested.

Enzyme cocktail 1 was also equally or more effective than the others in recovering the greatest

**Figure 1** Total cell recovery following dissociation of tumours with different enzyme cocktails. Mean ± s.e. provided for three or more determinations. Enzyme cocktail 1 (■); 2 (▩); 3 (□); 4 (▨).

**Table I** Cell types present following tumour dissociation with different enzyme cocktails. Mean ± s.e. provided for three or more determinations.

| | *Enzyme cocktail* | *% Tumour* | *% Macrophage* | *% Neutrophil* | *% Lymphocyte* |
|---|---|---|---|---|---|
| Col12 | 1 | 78 ± 3 | 20 ± 3 | 2 ± 0.3 | 1 ± 0.5 |
| | 2 | 63 ± 3[a] | 32 ± 3[a] | 3 ± 0.5 | 2 ± 0.5 |
| | 3 | 76 ± 2 | 20 ± 2 | 1 ± 0.3 | 3 ± 1 |
| | 4 | 71 ± 4 | 23 ± 2 | 4 ± 1 | 3 ± 0.7 |
| HEp3 | 1 | 64 ± 8 | 20 ± 1 | 13 ± 7 | 3 ± 1 |
| | 2 | 72 ± 12 | 12 ± 1[a] | 16 ± 12 | 1 ± 1 |
| | 3 | 61 ± 5 | 28 ± 2 | 10 ± 4 | 2 ± 1 |
| | 4 | 70 ± 7 | 14 ± 1[a] | 15 ± 8 | 2 ± 0.5 |
| A431 | 1 | 55 ± 6 | 24 ± 3 | 17 ± 6 | 4 ± 2 |
| | 2 | 10 ± 3[a] | 17 ± 4 | 71 ± 5[a] | 1 ± 1 |
| | 3 | 45 ± 7 | 20 ± 4 | 30 ± 12 | 4 ± 3 |
| | 4 | — | — | — | — |
| ME180 | 1 | 68 ± 10 | 26 ± 7 | 0.2 ± 0.1 | 6 ± 3 |
| | 2 | 58 ± 3 | 38 ± 1 | 2 ± 1 | 2 ± 1 |
| | 3 | 53 ± 10 | 37 ± 8 | 7 ± 3 | 2 ± 1 |
| | 4 | 50 | 46 | 4 | 1 |

[a]Indicates values which are significantly different ($P<0.05$) from those obtained with enzyme cocktail 1.

numbers of neoplastic cells from each tumour (Table I), although for Col12, HEp3 and ME180 tumours, the variation in tumour cell recovery was small when the four enzyme cocktails were compared. However, for A431 tumours, only enzyme cocktails 1 and 3 were able to release appreciable numbers of tumour cells. The use of enzyme cocktail 4 with A431 tumours consistently resulted in cell preparations containing large numbers of damaged and lysed cells and few recognizably intact tumour cells.

*Clonogenic assays*

Following dissociation with enzyme cocktail 1 an enhanced plating efficiency was observed for A431 ($P<0.05$ for enzyme cocktail 3; $P<0.025$ for enzyme cocktails 2 and 4) and ME180 tumours ($P<0.05$ for enzyme cocktail 3; $P<0.025$ for enzyme cocktail 4) (Figure 2a). However, based on plating efficiency alone, no clear distinction could be demonstrated for HEp3 tumours dissociated with enzyme cocktails 1–4. For Col12 tumours, dissociation with enzyme cocktails 3 and 4 resulted in slightly increased plating efficiencies as compared to the results obtained with cocktails 1 and 2 ($P<0.05$).

The inclusion of up to $10^4$ lethally irradiated (HR) tumour cells in the clonogenic assay produced variable results (Figure 2b). No change in plating efficiency could be demonstrated for ME180 cells isolated by different enzyme techniques and plated with and without HR cells (Figure 2a *vs.* 2b). Conversely, the inclusion of HR cells improved the plating efficiency of A431 cells isolated using each of the enzyme cocktails. The plating efficiencies of each tumour were corrected to account for the varying proportions of tumour cells recovered with each enzyme cocktail (Figure 3). For Col12 and HEp3 tumours the corrected plating efficiencies were similar for all enzyme cocktails tested. However, for A431 tumours, dissociation with enzyme cocktail 2 resulted in an improved corrected plating efficiency relative to that observed with enzyme cocktails 1 or 3 ($P<0.05$). For ME180 tumours, the corrected plating efficiency after dissociation with enzyme cocktail 1 was similar to that of enzyme cocktails 2 and 3, but was increased as compared to that of enzyme cocktail 4 ($P<0.05$).

Each enzyme cocktail was also compared for its ability to release the greatest number of clonogenic cells $g^{-1}$ tumour (Figure 4). For Col12, A431 and ME180 tumours, enzyme cocktail 1 was markedly superior to the other cocktails tested ($P$ values ranged from $<0.05$ to $<0.0025$). However, for HEp3 tumours, comparable clonogenic cell yields were found when all four enzyme cocktails were compared.

*Tumour heterogeneity*

The data presented in Table I indicate that different proportions of host and tumour cells were

**Figure 2** Uncorrected plating efficiencies of tumours dissociated with different enzyme cocktails and plated in the absence (a) or in the presence (b) of lethally irradiated (HR) cells. Note the difference in the *y*-axis. Mean ± s.e. provided for 3 or more determinations. For intepretation of cross-hatching see **Figure 1** (footnote).

recovered using different enzyme dissociation techniques. This was most strikingly demonstrated in A431 tumours in which the percentage of tumour cells recovered ranged from 10% (for enzyme cocktail 2) to an average of 55% (for enzyme cocktail 1) ($P<0.01$). Concomitantly, the recovery of the various types of host cells associated with A431 tumours varied markedly. The relative proportions of macrophages remained constant when the four enzyme cocktails were compared. However, the number of neutrophils ranged from average values of 17% (for enzyme cocktail 1) to 71% (for enzyme cocktail 2) ($P<0.01$). While the changes in the other tumour models tested were nowhere near as extreme as those seen in the A431 tumours, differences in the proportions of host and tumour cells still were observed. Similar to the morphometric evaluations, FCM analysis of the cell populations obtained from each type of tumour using different dissociation techniques suggested that different proportions of $G_1$, S and $G_2M$ tumour cells were recovered (Table II). For example, aberrant DNA distribution profiles were especially prominent in ME180 tumours as indicated by the reduced yield of $G_1$ tumour cells following treatment with enzyme cocktail 4 and by the significant variation in numbers of S and $G_2M$ tumour cells among the four cell preparations analyzed.

The heterogeneity in cell recovery and plating efficiency which was described for ME180, A431, Col12 and HEp3 tumours was also observed when tumours derived from two sublines of HEp3 cells were compared. The HEp3 KSCl.1 and HEp3SC$_2$LM cell lines originated from HEp3 cells passaged in a chick embryo and from a HEp3 lung metastasis in a nude mouse, respectively, and were chosen to determine the fidelity of parental

**Figure 3** Plating efficiencies of tumours corrected for varying proportions of neoplastic cells recovered following dissociation with different enzyme cocktails. For interpretation of cross-hatching see **Figure 1** (footnote).

**Figure 4** Total number of clonogenic cells recovered per g of tumour following dissociation of the tumour with different enzyme cocktails. For interpretion of cross-hatching see **Figure 1** (footnote).

**Table II** The percentage of $G_1$, S and $G_2M$ cells recovered from each tumour following dissociation with each enzyme cocktail.

| | | $G_1$ | $S$ | $G_2M$ |
|---|---|---|---|---|
| Col12 | (#1) | 64.3 | 28.9 | 6.8 |
| | (#2) | 41.4 | 43.7 | 14.9 |
| | (#3) | 40.3 | 37.4 | 22.3 |
| | (#4) | 64.1 | 32 | 3.9 |
| Hep3 | (#1) | 65.5 | 18.9 | 15.6 |
| | (#2) | 88.6 | 7.1 | 4.3 |
| | (#3) | 64.8 | 24.4 | 10.8 |
| | (#4) | 78.2 | 18.5 | 3.3 |
| A431 | (#1) | 52.7 | 41.6 | 5.7 |
| | (#2) | 64.2 | 34.9 | 0.9 |
| | (#3) | 58.4 | 33.9 | 7.7 |
| | (#4) | 59.6 | 35.9 | 4.5 |
| ME180 | (#1) | 91 | 1.3 | 6.7 |
| | (#2) | 70.6 | 20 | 9.4 |
| | (#3) | 80.9 | 10 | 9.1 |
| | (#4) | 66.4 | 32.3 | 1.3 |

characteristics such as plating efficiency and percentage of tumour *versus* host cells, following dissociation with different enzyme techniques. The data provided in Table III show that the use of different enzyme cocktails results in a greater variation in both total cell recovery and plating efficiency for the HEp3 tumour sublines than was observed for the original tumour. In addition, the relatively constant proportions of neoplastic cells recovered from HEp3 tumours using different enzyme cocktails was not observed when the two sublines of HEp3 cells were analyzed. Variations in the proportions of different types of host cells recovered following different enzyme dissociations were also noted for the HEp3 tumour sublines. For example, while the proportion of neutrophils in HEp3 tumours separated using different enzyme cocktails ranged from 10–16%, in the tumours derived from different HEp3 sublines, the proportion of neutrophils recovered ranged from 19% to 84% depending upon which enzyme cocktail was used for tumour dissociation.

**Table III** Comparison of HEp3 parental and HEp3 sub-line tumour characteristics. Mean±s.e. provided for three or more determinations.

| | *Enzyme cocktail* | *Cells* ($\times 10^7$) $g^{-1}$ *tumour* | *P.E.* ($\times 10^{-2}$) | % *Tumour* | % *Macrophage* | % *Neutrophil* | *Lymphocyte* |
|---|---|---|---|---|---|---|---|
| HEp3 | 1 | 3.9±1.1 | 5.5±1.8 | 64±8 | 20±1 | 13±7 | 3±1 |
| | 2 | 2.3±0.5 | 5.0±2.5 | 72±12 | 12±1 | 16±12 | 1±1 |
| | 3 | 2.1±1.2 | 9.6±1.7 | 61±5 | 28±2 | 10±4 | 2±1 |
| | 4 | 2.3±0.7 | 8.5±4.7 | 70±7 | 14±1 | 15±8 | 2±0.5 |
| HEp3 | 1 | 5.0 | ND[a] | 34 | 7 | 58 | 10 |
| KSC1.1 | 2 | 4.4 | ND[a] | 55 | 8 | 36 | 7 |
| | 3 | 4.3 | ND[a] | 10 | 2 | 84 | 4 |
| | 4 | 8.6 | ND[a] | 52 | 2 | 44 | 2 |
| HEp3 | 1 | 4.7±1.1 | 6.1±2.7 | 43±5 | 23±8 | 30±10 | 3±0.5 |
| $SC_2LM$ | 2 | 3.2±0.3 | 11.8±3.2 | 56±6 | 19±10 | 19±4 | 6±3 |
| | 3 | 1.9±0.2 | 6.8±2.7 | 34±5 | 17±1 | 49±5 | 3±1 |
| | 4 | 4.8±2.0 | 11.0±2.9 | 65±7 | 13±2 | 22±7 | 3±1 |

[a]Not determined.

### Discussion

These studies have demonstrated that marked variation in total cell recovery, plating efficiency and host *versus* tumour cell ratio can be observed when the same tumour is dissociated with different enzyme cocktails. In addition, significant variation in these parameters exists among the different epidermoid tumours tested. Although for each tumour tested, enzyme cocktail 1 consistently produced total cell yields which were two-fold greater than those observed with the other enzyme preparations, the total cell yield among the four tumours also varied by a factor of 2. With all enzyme cocktails tested, some pieces of tumour remained undissociated. However, preliminary experiments with enzyme cocktail 1 indicated that incubation times of more than 1 h did not result in the release of significantly more cells. The effect of variation in incubation time on the cell recovery with other enzyme cocktails has yet to be determined. The use of different enzyme cocktails also modified the corrected plating efficiencies of the tumours tested. These results were somewhat unexpected. If the variation in the effect of different enzyme cocktails was limited to differences in total number of cells recovered, one would expect that the plating efficiency of these cells, when corrected for the number of tumour cells present in a given cell suspension, would have been equivalent. However, our results indicate that the use of different enzyme dissociation techniques modifies the plating efficiency. For example, under optimum conditions (Figure 3) the plating efficiency of Co112 and HEp3 tumours is relatively constant for all enzyme preparations. However, in the case of ME180 tumours, a significant decrease in plating efficiency was observed when tumours were dissociated with enzyme cocktail 4, while for A431 tumours, cocktail 2 was superior. In these studies, the same *in vitro* plating technique was used for all tumours. It is not clear whether this technique affords optimum growth potential for all of the tumour and enzyme combinations studied. In addition, current studies do not allow us to distinguish between the possibilities that for ME180 tumours, in particular, these observed differences are due to the enzyme cocktails recovering quantitatively fewer tumour cells which are capable of forming colonies in agar or that these enzyme preparations are themselves cytotoxic to clonogenic cells derived from this tumour. While it is possible that the plating efficiencies might be improved by a brief holding period to allow for the repair of enzyme-induced damage, such a technique would not be suitable for use in studies which assessed the therapeutic effectiveness of radiation or chemotherapeutic agents on tumour cell survival.

While the above variations in plating efficiency were observed for tumours plated in the presence of lethally irradiated (HR) cells, it was noted that the choice of enzyme dissociation technique was able to alter the requirements for HR cells in the clonogenic assay (Figure 2). HR cells are thought to improve the plating efficiency of most tumours by providing an as yet unidentified source of growth factors (Courtenay, 1983) or by increasing the oxygen consumption in the culture, thereby lowering the oxygen tension, a condition which is regarded by some investigators as favouring tumour growth *in vitro* (Walls & Twentyman, 1985). In these studies, the addition of up to $10^4$ HR cells to the cultures did not uniformly improve the plating efficiency of the tumours studied. For ME180 tumours, no difference in plating efficiency was observed when cells were plated with or without HR cells. Conversely, for A431 tumours, (i) the addition of HR cells improved all plating efficiencies, and (ii) resulted in all plating efficiencies being approximately equal. For Co112 and HEp3 tumours, only the plating efficiency of cells obtained using enzyme cocktails 1 and 2, or 1, respectively, were improved by the addition of HR cells. It remains to be determined whether an increase in the number of HR cells added to the cultures would have improved the plating efficiency of tumours initially unaffected by the addition of HR cells. Nonetheless, these results suggest that for some tumours, a given enzyme cocktail may modify a tumour cell's ability to respond to autocrine growth factors, or may impair the ability of the irradiated cells themselves to produce and release these factors.

The experiments which analyzed the types of cells present in the cell suspensions obtained following tumour dissociation demonstrate that different enzyme preparations can recover different proportions of host and tumour cells. Siemann *et al.* (1981) have demonstrated that murine tumours can contain appreciable numbers (30–60%) of host cells. Also using murine tumours it has been shown that the recovery of host cell populations from a tumour can be altered by the use of different enzyme dissociation techniques (Russell *et al.*, 1976; Siemann *et al.*, 1981). In our experiments, the percentage of tumour cells in a given cell preparation was relatively constant for HEp3, Co112 and ME180 tumours (Table I). However, recovery of A431 tumour cells varied widely when the results obtained following different enzyme dissociation techniques were compared (Table I). When enzyme cocktail 4 was used, few intact tumour or host cells could be identified, whereas when this same enzyme cocktail was used with the other tumours, cell harvests of 50–70% tumour cells were obtained (Table I). The FCM analysis of the cell suspensions obtained with different enzyme cocktails also supports the hypothesis that different populations of cells can be obtained from the same tumour specimen when different enzyme cocktails are used.

In summary, the present experiments have demonstrated that the choice of an enzyme dissociation technique can modify factors such as host and neoplastic cell yields from human tumour xenografts and point to the need for careful characterization of each tumour system before it can be used to address questions of basic human tumour biology or response of tumours to therapy. In our studies, tumour cell plating efficiency expressed as the number of clonogenic cells $g^{-1}$ tumour was the endpoint used to assess the efficacy of the four different enzyme dissociation techniques. Using this criteria, enzyme cocktail 1 was found to be superior to the other enzyme preparations tested. However, these results should be interpreted as applying particularly to these tumours and this biological endpoint. It is possible, as suggested by Steel and Peckham (1980), that other dissociation techniques would provide superior results if different tumours or different endpoints are used. For example, xenografted tumours derived from sarcomas may require a different battery of enzyme to result in adequate tumour dissociation. Similarly, if one were to study a different biological endpoint, e.g., the amplification of epidermal growth factor receptors on A431 tumours following different *in vivo* manipulations (Stoscheck & Carpenter, 1983), one might observe enzymes found most effective in the present investigations to be ineffective at preserving the expression of these receptors following tumour dissociation. Similarly, different treatments such as chemotherapy or irradiation may have differential effects on the host *versus* neoplastic cell proportions of a tumour. In such a case, a determination of the relative proportions of host *versus* neoplastic cells which persist in a tumour following treatment would be necessary in order to accurately estimate the corrected tumour cell plating efficiency. These findings suggest that basic studies aimed at characterizing the response of human xenograft material to different dissociation techniques are necessary to ensure that this aspect of the experimental technique has been optimized for the tumours under investigation.

This work was supported by NIH grants CA-11051, CA-11198, CA-38637 and CA-36858. MJAT is the recipient of a fellowship from the Alberta Heritage Foundation for Medical Research. The authors wish to thank Dr Peter C. Keng for performing the flow cytometric analysis, K. Alliet, S. Curnick, K. Faro, B. King and K. Wolf for technical assistance, and B. Granger for preparation of the manuscript. The support of the Animal Tumor Research/Xenograft Facility of the University of Rochester Cancer Center is gratefully acknowledged.

### References

COURTENAY, D. (1983). The Courtenay clonogenic assay. In *Human Drug Sensitivity Testing in vitro*, Dendy & Hill (eds) p. 103. Academic Press: London.

ENGELHOLM, S.A., SPANG-THOMSEN, M., BRUNNER, N., NOHR, I. & VINDELOV, L.L. (1985). Disaggregation of human solid tumours by combined mechanical and enzymatic methods. *Br. J. Cancer*, **51**, 93.

FOGH, J. & GIOVANELLA, B.C. (1977). *The Nude Mouse in Experimental and Clinical Research*. Academic Press: New York.

FRIED, J. & MANDEL, M. (1979). Multi-user system for analysis of data from flow cytometry. *Comput. Programs Biomed.*, **10**, 218.

GIARD, D.N., AARONSON, S.A., TODARO, G.J. & 4 others (1973). *In vitro* cultivation of human tumors: establishment of cell lines derived from a series of solid tumors. *J. Natl Cancer Inst.*, **51**, 417.

MACH, J.-P., CARREL, S., MERENDA, C., SORDAT, B. & CEROTTINI, J.-C. (1974). *In vivo* localization of radiolabelled antibodies to carcinoembryonic antigen in human colon carcinoma grafted into nude mice. *Nature*, **248**, 704.

NG, C.E. (1978). The density distribution of cells from a mouse tumor: effects of growth conditions and irradiation. *Ph.D. Thesis, University of Western Ontario*.

RASEY, J.S. & NELSON, N.J. (1980). Response of an *in vivo-in vitro* tumour to X-rays and cytotoxic drugs: effect of tumour disaggregation method on cell survival. *Br. J. Cancer*, **41**, Suppl. IV, 217.

RUSSELL, S.W., DOE, W.F., HASKENS, R.G. & COCHRANE, C.G. (1976). Inflammatory cells in solid murine neoplasms. I. Tumor disaggregation and identification of constituent inflammatory cells. *Int. J. Cancer*, **18**, 322.

SIEMANN, D.W., LORD, E.M., KENG, P.C. & WHEELER, K.T. (1981). Cell populations dispersed from solid tumours and separated by centrifugal elutriation. *Br. J. Cancer*, **44**, 100.

STEEL, G.G. & PECKHAM, M.J. (1980). Human tumour xenografts: a critical appraisal. *Br. J. Cancer*, **41**, Suppl. IV, 133.

STOSCHECK, C.M. & CARPENTER, G. (1983). Biology of the A-431 cell: a useful organism for hormone research. *J. Cellular Biochem.*, **23**, 191.

SYKES, J.A., WHITESCARVER, J., JERNSTROM, P., NOLAN, J.F. & BYATT, P. (1970). Some properties of a new epithelial cell line of human origin. *J. Natl Cancer Inst.*, **45**, 107.

TOOLAN, H.W. (1954). Transplantable human neoplasms maintained in cortisone-treated laboratory animals. H.S. No. 1; H.Ep. No. 1; H.Ep. No. 2; H.Ep. No. 3; and H.Emb.Rh. No. 1. *Cancer Res.*, **14**, 660.

TWENTYMAN, P.R., BROSON, J.M., GRAY, J.W., FRANKO, A.J., SCOLES, M.A. & KALLMAN, R.F. (1980). A new mouse tumor model system (RIF-1) for comparison of end-point studies. *J. Natl Cancer Inst.*, **64**, 595.

WALLS, G.A. & TWENTYMAN, P.R. (1985). Cloning of human lung cancer cells. *Br. J. Cancer*, **52**, 505.